# Quality of Life in Persons with Intellectual Disabilities and Mental Health Problems: An Explorative Study

**DOI:** 10.1155/2013/491918

**Published:** 2013-02-05

**Authors:** Filip Morisse, Eleonore Vandemaele, Claudia Claes, Lien Claes, Stijn Vandevelde

**Affiliations:** ^1^Psychiatric Centre Dr. Guislain, Sint-Juliaanstraat 1, 9000 Ghent, Belgium; ^2^Den Dries Service Center, Kramershoek 39, 9940 Evergem, Belgium; ^3^Faculty of Education, Health and Social Work, University College Ghent, Voskenslaan 362, 9000 Ghent, Belgium; ^4^Department of Orthopedagogics, Ghent University, Henri Dunantlaan 2, 9000 Gent, Belgium

## Abstract

The field of intellectual disability (ID) is strongly influenced by the Quality of Life paradigm (QOL). We aimed at investigating whether or not the QOL paradigm also applies to clients with ID and cooccurring mental health problems. This paper aims at stimulating a debate on this topic, by investigating whether or not QOL domains are universal. Focus groups with natural and professional network members were organized to gather qualitative data, in order to answer two questions: (1) Are the QOL dimensions conceptualized in the model of Schalock et al. applicable for persons with ID and mental health problems? (2) What are indicators relating to the above-mentioned dimensions in relation to persons with ID and mental health problems? The results offer some proof for the assumption that the QOL construct seems to have universal properties. With regard to the second question, the study revealed that the natural and professional network members are challenged to look for the most appropriate support strategies, taking specific indicators of QOL into account. When aspects of empowerment and regulation are used in an integrated manner, the application of the QOL paradigm could lead to positive outcomes concerning self-determination, interdependence, social inclusion, and emotional development.

## 1. Introduction

The field of intellectual disability (ID) is strongly influenced by the Quality of Life paradigm (QOL), from a research, a practice-based, and a policy-oriented perspective [1–5]. This QOL framework supports the equality of persons, which is reflected in concepts such as self-determination, emancipation, inclusion, and empowerment. In daily practice however, in which concepts are translated into tangible actions, professionals are often confronted with difficulties to apply these QOL principles. This seems especially true when working with specific populations, including persons with ID and mental health problems. The application of QOL principles, which should—in ideal conditions—lead to positive outcomes with regard to social participation, independence, and well-being [6], seems to be at risk, as accounts from professionals indicate that empowerment is sometimes replaced by actions solely aimed at “controlling,” “dominating and excluding” clients with ID and cooccurring mental health problems.

This paper aims at stimulating a debate on this topic, by investigating whether or not QOL domains are universal and applicable to people with ID and mental health problems. Although the cooccurrence of mental health problems can be described as an important issue in the field of ID research and practice, there have not been many studies tackling the application of the QOL paradigm in this specific population. 

### 1.1. Quality of Life (QOL)

The construct of QOL has been widely applied in the field of ID and implies principles of emancipation and inclusion. Initially, the assessment of QOL was approached from multiple perspectives, resulting in over 1,243 measures reported in the QOL literature by the mid-1990s [7]. The current approach to the measurement of QOL can be characterized by the following:

- its multidimensional nature involving core domains and indicators;
- the use of methodological pluralism that includes the use of subjective and objective measures;
- the incorporation of a systems perspective that captures the multiple environments impacting people at the micro-, meso-, and macrosystems levels; and
- the increased involvement of persons with ID in the design and implementation processes [5, 8].

In this study, we adopt the following definition of individual-referenced QOL [9]:

*“Individual QOL is a multi-dimensional phenomenon composed of core domains that are influenced by personal characteristics and environmental variables. These core domains are the same for all people, although they may vary in relative value and importance. QOL domains are assessed on the basis of culturally sensitive indicators.”*

The QOL construct consists of the eight domains that have been validated in a series of cross-cultural studies [6, 10–12]. These eight domains are personal development and self-determination (that reflect a person's level of independence); interpersonal relations, social inclusion, rights (that reflect a person's social participation); emotional, physical, and material well-being. The QOL literature does not define a hierarchy amongst those domains nor does it specify cause and effect relations amongst them [8]. QOL indicators are QOL-related perceptions, behaviours and conditions that operationally define each QOL domain.

### 1.2. Persons with ID and Mental Health Problems: Terminology, Prevalence, and Support Needs

The prevalence of psychiatric disorders in people with ID is higher as compared to the general population [13]. Epidemiological studies estimate the prevalence of behaviour problems and psychiatric disorders amongst individuals with ID at 30–50% [14]. The coexistence and interference of the symptoms of both ID and mental health problems are multiple and complex. This is, for instance, reflected in the lack of an internationally recognized and uniform definition and terminology [14]. Throughout the literature, concepts including “dual diagnosis,” “cooccurring disorders,” “mental health problems,” “mental health needs,” “behavioural problems,” “behavioural disorders,” “conduct disorders,” and “challenging behaviour” are used. In this paper, it was chosen to use the term “mental health problems” in persons with ID. By doing so, we include both “behavioural problems” or “challenging behaviour” [15] as well as psychiatric disorders as defined in currently used manuals, such as the DSM-IV or the ICD-10.

Persons with ID and mental health problems might be amongst the most vulnerable groups of people in our society [13]. Up until now, the medical framework has been very dominant in supporting persons with ID and mental health problems. According to some authors, this is due to the complexity of physical, emotional, and behavioural issues [13, 16]. Under impetus of this tendency, traditional mental health services have focused on establishing special healthcare units. Despite the deinstitutionalization movement, community-based services for people with ID and mental health problems are still scarce [17]. This observation could explain why it is more difficult to make the QOL paradigm operational for this population and its care system than for support systems in which concepts as inclusion and participation are more obvious. 

### 1.3. Quality of Life in Persons with ID and Mental Health Problems

Despite a high number of studies on QOL in people with an ID, few empirical studies specifically tackled QOL of people with ID and mental health problems. Yet, the coexistence of ID and mental health problems can have far-reaching effects on the person's daily functioning and QOL. In this respect, adequate support is a challenge, as Došen and Day [18] argue for an integration of medical, psychotherapeutic, behavioural, cognitive, milieu, and pedagogical treatment methods to enhance QOL. Because of this complexity, the application of the QOL paradigm is not self-evident, although there seems to be consensus about the fact that the same domains are relevant for all persons, including this specific subpopulation. As very few studies exist on QOL for people with an ID and mental health problems, we aimed to explore how the eight-domain QOL construct by Schalock et al. [6] can be operationalized for persons with ID and mental health problems. This leads to the following research questions:

1. Are the QOL dimensions conceptualized in the model of Schalock et al. [6] applicable for persons with ID and mental health problems?
2. What are indicators relating to the above-mentioned dimensions in relation to persons with ID and mental health problems?

## 2. Method

### 2.1. Participants

The study [19] took place in Flanders, the Dutch-speaking Northern part of Belgium. A partnership amongst three organizations in the support system for people with ID and mental health problems was developed. The Flemish support systems consist of two distinct care systems, which evolved separately: mental health care on the one hand and the care and support system for people with ID on the other hand. Historically, people with ID were supported within mental health care settings (starting from the idea that “intellectual disability” was a mental health problem), but from the 1960s onwards, a separate support system for people with disabilities has emerged. This shift, however, resulted in people with ID and mental health problems frequently falling “between the gaps” [20]. While mental health care stated that “people with disabilities” have to be supported within the care system of people with disabilities, this latter care system claimed that the treatment of people with mental health problems is the responsibility of mental health care [21]. Nowadays, professionals in both systems attempt to collaborate within continuums of care, although both systems still exist in their own right. This study tried to involve caregivers of both care/support systems. On the one hand, two observation and treatment units for people with ID within mental health care participated in the study. On the other hand, a unit for people with ID and mental health problems within a service center for people with ID was involved. 

In these services, participants were selected by purposeful sampling [22] and were contacted by the employees of these organizations. The sample consisted of persons from the natural network (*n* = 7) and representatives of the professional network (staff members) (*n* = 10) of people with ID and mental health problems. To achieve a heterogeneous sample of participants, a number of parameters were taken into account: gender, age, place of residence of the client (mental health care or support system for people with ID), type of mental health problems, and level of ID. 

### 2.2. Instruments

Focus groups were organized to gather qualitative data. The first focus group consisted of four mothers, two fathers, and one stepmother, who were all closely involved with their family members with ID. The second focus group consisted of professionals who were employed in the three facilities represented in this research: three staff members of both the psychiatric centers and four of the unit for people with ID and mental health problems within a service centre for people with ID. 

The selection of the professional workers/staff was based on age, gender, years of experience (from 1 up to 30 years of experience), and their level of education. The staff members of the psychiatric centers were psychiatric nurses or educational specialists. These of the service centre for people with ID were educational specialists and one social worker.

### 2.3. Procedure

As a first step, the purpose of the research was explained to the participants, who were also asked to sign an informed consent form. The focus group discussions took about 90 minutes and were led by the second author of this paper, who was assisted by the fourth and fifth authors of this paper. Each focus group was organized twice. In the first focus group participants were asked to brainstorm and reflect on how they consider “Quality of Life” in general and for their family member/client in particular: “which things are important to be able to talk about a quality life for people with ID and mental health problems and for your family member in particular?” In the second focus group, the data from the first focus group were grouped into the eight domains of the QOL construct as developed by Schalock et al. [6] and were conceptualized in indicators, which turn out to be important for the research population. 

### 2.4. Analysis

The four focus groups were audio- and video-taped and were literally transcribed. Two of the authors independently read these transcripts and identified domains/categories and indicators/themes, which guaranteed the interrater reliability. Structuring and clustering the results were primarily based on the QOL construct of Schalock et al. [6]. Statements obtained in the first focus groups were classified in those eight domains (personal development, self-determination, interpersonal relations, social inclusion, rights, emotional well-being, physical well-being, and material well-being). In the second focus groups, the participants were asked to operationalize the indicators corresponding with the eight domains. 

## 3. Results

The *first research question* investigates whether the QOL dimensions, which are conceptualized in the model of Schalock et al. [6], are applicable for persons with ID and mental health problems. The results show that the participants mentioned aspects of all eight domains (personal development, self-determination, interpersonal relations, social inclusion, rights, emotional well-being, physical well-being, and material well-being) as a response to the general question of the first focus groups.  Table 1 reports how frequently professional and natural network members talked about aspects from the eight domains. This reflects which domains received more or less attention. 

The domains of “emotional well-being,” “interpersonal relations,” “self-determination,” and “social inclusion” were mentioned most often. “Self-determination” and “interpersonal relations” were more frequently cited by professionals, while “social inclusion,” appeared to be an important domain for families. “Emotional well-being” was mentioned most frequently by both natural and professional network members. Particularly, the domains of  “rights” and “physical well-being” received less attention. In addition, compared to “emotional well-being,” “interpersonal relations,” “social inclusion” and “self-determination,” also “material well-being” and “personal development” were mentioned less often. 

The *second research question* explores which indicators are related to the mentioned domains, specifically in relation to persons with ID and mental health problems.  Table 2 shows which indicators were mentioned in relation to a particular domain. The domains that were discussed most extensively (“emotional well-being,” “interpersonal relations,” “self-determination,” and “social inclusion”) and their related indicators are further elaborated in the following section. 

### 3.1. Domain Self-Determination

#### 3.1.1. Freedom of Choice

Both natural network and professional staff members indicated that it is important to enable persons with ID and mental health problems to choose as much as possible, albeit to the extent they can handle. In their opinion, offering a limited number of choices seems to be the best option in this respect. Giving too many choices is usually confusing and too abstract, which can lead to stress and anxiety.

*“I have already noticed, if you offer a limited number of choices, she will choose. […] But it has to be limited, otherwise she is not able to manage it anymore.” (Member of the social network)*

#### 3.1.2. Freedom

Professional network members indicated that the QOL of persons with ID and mental health problems is highly impacted by measures of restricted freedom. Especially in the transition to adulthood, persons suddenly receive more freedom, which may cause problems. Sufficient support and guidance is necessary to support people in coping with this “newly gained” freedom.

*“If people's verbal possibilities are sufficient and you talk about freedom profoundly, they actually feel locked up. […]. They go out a lot and do many things, but they rarely go on one's own, which give them a feeling of being locked up and restraint. In our opinion, for certain people, the quality of life is better when they live in such a regimen, but it is not their opinion.” (Professional in the care system for people with ID)*

#### 3.1.3. Boundary/Limitation

Persons with ID and mental health problems seem to have difficulties with imposing limits on themselves. One of the professional workers defined this behavior as “bottomless.” Nutrition, for example, seems sometimes hard to restrain.This may be caused by stress and restlessness on an emotional level. The refuge into food abuse could be seen as compensational behavior of an emotional unbalance. According to the network members, and professional staff, this seems also true for financial matters such as “buying behavior”. Therefore, persons with ID and mental health problems directly and indirectly ask to apply external boundaries, which provide safety and structure. Lack of insight into the consequences of their actions may account for this need to external control.

*“We also need this [restrictions], but for ourselves, we do this intrinsically, we restrict ourselves and we consider. They [people with ID and mental health problems] do not have those skills and many things are taken over […].” (Professional in the care system for people with ID)*

### 3.2. Domain Interpersonal Relationships

#### 3.2.1. Social Contact, Social Network

Persons with ID and mental health problems seem to have a great need for social interaction, just like people without ID have. In practice, it is not obvious, however, to build and maintain relationships. The social network of these people is mostly limited to family, professional staff members and fellow clients when residing in support or care services. Network members indicated that they perceived their sons or daughters to be more satisfied with the relationships they have with people of thesame intellectual level.

*“(…) Because they ask for it. They ask: “Search me a friend!.” So those people also know that their world is very small and that they are constantly looking for new contacts. It is frustrating if you do not find those people. And if you meet someone one day it often the case they, who have social disabilities to lose their friends again. Those people lose interest.” (Professional in the care system for people with ID)*

#### 3.2.2. Professional Guidance

According to professional staff members, the relationship between the client and the support worker is an essential aspect of the QOL of persons with ID and mental health problem. An important issue in this respect is the large staff turnover within facilities.

*“To me a major quality-killer is the high turnover within facilities, which to me, is a highly underestimated factor.” (Professional in the care system for people with ID)*

#### 3.2.3. Relationships

Clients appear to have a strong need for a long-lasting relationship. This can be explained from the desire to live “a normal life”. The accounts of professionals and natural network members underscore that persons with ID and mental health problems want to have a similar life as anyone else. In most cases, however, this is not always possible with important consequences for their QOL.

### 3.3. Domain Social Inclusion

#### 3.3.1. “A Normal Life”

Both caregivers and family members mention that persons with ID and mental health problems very often want to follow the example set forth by people without ID. Many of them long to having a partner, a job, a house, children, and friends.

*“Take warning from the standards in society. Everybody marries, everybody get children… And we are just here, we do not have a girlfriend and we could hardly have a beloved because we should be supported in an institution.” (Professional in mental health care)*

#### 3.3.2. To Be Accepted by Others

Many persons with ID and mental health problems deal with a low self-image, as a result of, for example, experiences of failing and difficulties encountered in their environment. Family members indicate the importance of having a feeling of acceptance and of “belonging” somewhere. Because persons with ID and mental health problems often “drop out” in social activities, it is important to make sure that people feel included and accepted.

### 3.4. Domain Emotional Well-Being

#### 3.4.1. Proximity

The proximity of caregivers and family members is important for the emotional well-being of persons with ID and mental health problems. This need could be attributed to the emotional restlessness that persons with ID often experience. Being surrounded by persons on whom to fall back seems to offer the necessary safety and security.

#### 3.4.2. Structure

Family members often emphasized that persons with ID and mental health problems benefit from a structured life. Similar to proximity, structure offers a sense of certainty and predictability. One of the parents stated that structure needs to be fine-tuned with respect to the personal needs of the client.

*“Structure which is considered to be “normal,” is not the structure that for instance my daughter needs. When you presents “the normal structure” to them, they try to wriggle, but it do not go “well”. It is very hard to imagine in the structure she needs for me as well (…).” (Natural network member)*

#### 3.4.3. Appreciation, Positive Attention, Confirmation, and To Be Taken Seriously

Caregivers and family members experience that the self-image of these people is positively affected when they feel appreciated and found useful by others. One of the mothers communicated the distressing point that people do not listen to her daughter, which results in a declining self-image. Paying attention to the strengths instead of the limitations is an important aspect to improve one's QOL.

#### 3.4.4. Respecting Their Own Pace

An important issue in the support of people with ID and additional mental health problems is to take into account the pace of the client. Often, people are confronted with too much pressure and too high expectations, which they cannot fulfill.

*“She could even not manage the work in the sheltered workplace because of the pressure she experienced. Now she goes to a day care centre. She works on her own tempo. She works with people who accept her and she do not experience pressure. It goes well.” (Natural network member)*

A quick accumulation of incidents has to be avoided. People need some time to cope with changes, problems, and incidents; time to get used, to adapt, and to find a way to cope, with or without support of family and/or caregivers. 

#### 3.4.5. Peaceful Time and Having an Overview

Chaos is a source of emotional restlessness and behavioral problems. Having an overview of what the day will consist of may support persons with ID and mental health problems.

*“In our organization, it is intrinsically united with their problems that they function on an emotional level in which they are still looking for safety which they do not find because they had a “wrong” bond before. Thus emotionally, they struggle for independence which they could never manage. They never experience “peace” or satisfaction…” (Professional in the care system for people with ID)*

#### 3.4.6. Watch Out for Overdemanding (=Asking Too Much)/Be Careful for Overcharge

Persons with ID in general and people with additional mental health problems in particular are regularly overdemanded, because of the discrepancy between the emotional and intellectual level of development. Over-demanding often results in mental health and behavioral problems. 

#### 3.4.7. Affection, Sociability, and Love

Persons with ID and mental health problems have a strong need for affection, sociability, love, acceptance, security, and safety.

#### 3.4.8. Medication

The positive effects of medication on the well-being of people may not be underestimated but only in a proportioned and considerate way. 

## 4. Discussion

The aim of this study was twofold. First we wanted to evaluate the relevance of the QOL dimensions as conceptualized in the model of Schalock et al. [6] for persons with ID and mental health problems according to family members and support workers. Second, we wanted to explore specific indicators related to the eight dimensions in relation to persons with ID and mental health problems. We conducted this study based on the eight-domain QOL conceptual model that has both etic (universal) and emic (cultural bound, related to specific life events or circumstances) properties [10]. 

In regard to the first question, this study confirms the relevance of the eight-domain conceptual QOL model. All domains were quoted spontaneously, which argues for the multidimensionality and universality of the construct. As all domains were reported in the focus groups, the eight-domain conceptual model is a valid model in QOL-assessment for persons with ID and mental health problems. Nevertheless, some domains were more quoted than others. The most common domains reported by professional workers were emotional well-being, interpersonal relationships, and self-determination. The domains reported most by network members were emotional well-being, social inclusion and interpersonal relationships. These results confirm the assumption that QOL may vary in relative value and importance. The relative importance of the domain emotional well-being in persons with ID and mental health problems can be explained by the vulnerability in emotional (and not only intellectual) development. People with ID and mental health problems are at risk because of the discrepancy between cognitive and emotional development [14]. Because the environment of people with ID predominantly addresses the easily perceptible cognitive development instead of the lower and masked emotional development, there is a risk to overestimate and overcharge people with ID. 

With regard to the second question, we evaluated how family members and support workers operationalize the different domains for people with ID and mental health problems. This part of the study revealed some interesting and creative responses which gave on the one hand insight in the specificity of this population and on the other hand offered some clues for support strategies. On the level of self-determination family members and support workers argue for own—but limited—choices. Another important observation is that the clear plea for freedom does not conflict with a certain amount of regulation. Furthermore, indicators on interpersonal relationships and social inclusion (social contacts, social network, support, integration, and participation) turn out to be less specific. Finally, the domain on emotional well-being was indicated most. Its interpretation in indicators (e.g., nearness, structure, positive attention, respecting own pace, watching out for over-demanding/overcharge) encourages reflection and needs to be considered as needs in the support plans of those vulnerable clients. 

The authors put forward two major implications to the field from the data reported in this paper. 

First, the QOL construct has universal properties and is on the level of domains the same for all people. This framework supports the equality of persons, which is reflected by concepts including self-determination, emancipation, inclusion and empowerment. 

Second, the presented challenges and difficulties with regard to the QOL of persons with ID and mental health problems clearly illustrate the difficult task natural and professional network members have to fulfil when supporting their family members and clients. The fact that it is not evident to cope with these challenges may lead to a wrong application of QOL principles, albeit with the best intentions. We would like to discuss two potentially harmful consequences that—in our opinion—can be situated on a continuum of extreme control and elimination of all risks on the one hand and a “laisser faire, laisser passer” attitude on the other hand. 

The concept of “duty of care” as expressed by many service providers is often used as a rationale for eliminating risks and therefore inhibits a person-centered approach [23]. This leads to a “bounded empowerment” where clients are supported in independence as long as it fits within the boundaries of health and safety [24]. To the authors' view, an integrative support paradigm offers a framework to consider the concepts of person-centered approach with opportunities for “risk-taking” and “real” empowerment as essential elements of a holistic view on supporting clients with ID and mental health problems [25]. Support staff should reflect on the individual pathology discourse people are put in and the way in which this inhibits opportunities in making choices and having control [24]. Instead of questioning the relevance of the QOL domains in people with ID and mental health problems, it seems important to reflect on what is needed and what is working in the areas of QOL [26]. 

On the other hand, because of the importance of issues with regard to the social-emotional development, structure, control, and predictability may not be considered as negative “an sich”. On the contrary, these regulating measures may improve one's QOL. It goes without saying, however, that this may not be used as an excuse to take over all responsibilities of persons with ID and mental health problems. 

There are some limitations in this study. First, although this study was the result of a partnership between three organizations, the results of the focus groups cannot be generalized due to the limited sample size. Second, the clients' perspectives about their own QOL are not reported in this paper. They are part of another study and will be published in the future.

Based on the results of this study, we can conclude that the natural and professional network members are challenged to look for the most appropriate support strategies that lead to an improvement in the QOL of their family members or clients with ID and cooccurring mental health problems. There is, however, a real risk that the QOL principles are not properly applied, which could lead to an elimination of risks and the use of empowerment within very limited contexts on the one hand or a “laisser faire, laisser passer” attitude that lacks the necessary structure and predictability on the other hand. When both aspects of empowerment and control are used in an integrated manner, the application of the QOL paradigm could lead to positive outcomes concerning self-determination, interdependence, social inclusion, and emotional development. 

## Figures and Tables

**Table 1:** Number of statements organized within the eight domains of QOL by Schalock et al. [6] for professional and natural network members [19].

Domain	Professional workers (PW)	Network members (NM)	Total	Percentage of PW	Percentage of NM
Emotional well-being	19	35	54	35,2%	64,8%
Interpersonal relationships	14	10	24	58,3%	41,7%
Self-determination	14	7	21	66,6%	33,4%
Social inclusion	7	14	21	33,3%	66,7%
Material well-being	7	4	11	63,6%	36,4%
Personal development	2	7	9	22,2%	77,8%
Rights	6	3	9	66,7%	33,3%
Physical well-being	1	1	2	50%	50%

**Table 2:** Operationalization of QOL domains into indicators by professional and natural network members [19].

Domains	Operationalization by network members and professional workers
Personal development	Education on the personal level, work, self-image
Self-determination	Independency, freedom of choice, freedom, boundary/limitation
Interpersonal relations	Social contacts, contact with people with the same intellectual capacities, social network, professional support, partner
Social inclusion	A normal life, to be accepted by others, going out/trips
Rights	Tailored care, general rights, privacy, children
Emotional well-being	Proximity, structure, appreciation, positive attention, confirmation, to be taken seriously, respecting their own pace, rest and overview, watch out for over-demanding (= asking too much)/be careful for overcharge-Affection, sociability, love, medication, nutrition
Physical well-being	Attention of the physician, coherence between emotional and physical well-being
Material well-being	Private space for living, more staff, financial and material resources, responsibility for expenses, status
